# Risk of Head and Neck Cancer in Former Smokers by Subsite: A Multicenter Analysis From the INHANCE Consortium

**DOI:** 10.1002/ijc.70497

**Published:** 2026-04-21

**Authors:** Matheus de Abreu, Luiz Paulo Kowalski, Rossana Mendoza López, Christine Barul, Loredana Radoi, Ettore Bidoli, Jerry Polesel, Victor Wunsch‐Filho, Andrew F. Olshan, Jose Zevallos, Eva Negri, Valeria Edefonti, Beata Świątkowska, Dana Mates, Eleonora Fabianova, Jolanta Lissowska, Oxana Shangina, Paul Brennan, Tamas Pandics, Luigino Dal Maso, Hal Morgenstern, Zuo‐Feng Zhang, Karl Kelsey, Michael McClean, Carlo La Vecchia, Werner Garavello, Chu Chen, Stephen M. Schwartz, Heribert Ramroth, Volker Winkler, Gabriella Cadoni, Stefania Boccia, Hermann Brenner, Gypsyamber D'Souza, Neil Gross, Joshua Muscat, Mahsa Abedini, Michele Sassano, Paolo Boffetta, Mia Hashibe, Yuan‐Chin Amy Lee, Maria Paula Curado

**Affiliations:** ^1^ Epidemiology and Statistics Nucleus, International Research Center, A.C.Camargo Cancer Center São Paulo Brazil; ^2^ Department of Head and Neck Surgery and Otorhinolaryngology A.C.Camargo Cancer Center São Paulo Brazil; ^3^ Cancer Institute of the State of São Paulo (ICESP) São Paulo Brazil; ^4^ Univ Rennes, Inserm, EHESP, Irset (Institut de Recherche en santé, Environnement et Travail) Pointe‐à‐Pitre France; ^5^ INSERM UMR 1018, Centre for Research in Epidemiology and Population Health (CESP), Cancer Epidemiology, Genes and Environment Team Villejuif France; ^6^ Cancer Epidemiology Unit, Centro di Riferimento Oncologico di Aviano (CRO) IRCCS Aviano Italy; ^7^ Department of Epidemiology, Faculdade de Saude Publica Universidade de Sao Paulo São Paulo Brazil; ^8^ Gillings School of Global Public Health University of North Carolina Chapel Hill North Carolina USA; ^9^ Department of Otolaryngology University of Pittsburgh Pittsburgh Pennsylvania USA; ^10^ Department of Medical and Surgical Sciences University of Bologna Bologna Italy; ^11^ Department of Clinical Sciences and Community Health University of Milan Milan Italy; ^12^ Nofer Institute of Occupational Medicine Lodz Poland; ^13^ National Institute of Public Health Bucharest Romania; ^14^ Regional Authority of Public Health Banska Bystrica Slovakia; ^15^ Department of Cancer Epidemiology and Prevention, M.Sklodowska‐Curie National Research Institute of Oncology Warsaw Poland; ^16^ Cancer Research Centre Moscow Russia; ^17^ International Agency for Research on Cancer Lyon France; ^18^ National Center for Public Health and Pharmacy Budapest Hungary; ^19^ Department of Public Health Sciences, Faculty of Health Sciences Semmelweis University Budapest Hungary; ^20^ Department of Epidemiology, School of Public Health University of Michigan Ann Arbor Michigan USA; ^21^ Department of Environmental Health Sciences, School of Public Health University of Michigan Ann Arbor Michigan USA; ^22^ Department of Urology, Medical School University of Michigan Ann Arbor Michigan USA; ^23^ Department of Epidemiology, UCLA Fielding School of Public Health Los Angeles California USA; ^24^ Brown University Providence Rhode Island USA; ^25^ Boston University School of Public Health Boston Massachusetts USA; ^26^ Department of Otorhinolaryngology, School of Medicine and Surgery University of Milano‐Bicocca Milan Italy; ^27^ Fred Hutchinson Cancer Center Seattle Washington USA; ^28^ Heidelberg Institute of Global Health, University of Heidelberg Heidelberg Germany; ^29^ Istituto di Clinica Otorinolaringoiatrica, Universita Cattolica del Sacro Cuore Roma Italy; ^30^ Istituto di Clinica Otorinolaringoiatrica, Fondazione Policlinico Universitario A. Gemelli Rome Italy; ^31^ Section of Hygiene, Department of Life Sciences and Public Health Università Cattolica del Sacro Cuore Rome Italy; ^32^ Department of Woman and Child Health and Public Health Fondazione Policlinico Universitario A. Gemelli IRCCS Rome Italy; ^33^ German Cancer Research Center Heidelberg Germany; ^34^ Johns Hopkins Bloomberg School of Public Health Baltimore Maryland USA; ^35^ Department of Head and Neck Surgery, Division of Surgery The University of Texas MD Anderson Cancer Center Houston Texas USA; ^36^ Penn State College of Medicine Hershey Pennsylvania USA; ^37^ Stony Brook Cancer Center, Department of Family, Population and Preventive Medicine Stony Brook University Stony Brook New York USA; ^38^ Department of Epidemiology, Fielding School of Public Health University of California Los Angeles California USA; ^39^ Division of Public Health, Department of Family and Preventive Medicine University of Utah School of Medicine Salt Lake City Utah USA; ^40^ Huntsman Cancer Institute Salt Lake City Utah USA

**Keywords:** alcohol drinking, former smokers, head and neck neoplasms, smoking

## Abstract

This study investigated risk factors associated with HNC by subsite including oral cavity cancer (OCC), oropharyngeal cancer (OPC), and laryngeal cancer (LC) among former smokers in the International Head and Neck Cancer Epidemiology Consortium (INHANCE). Case‐control study including former smokers from the pooled INHANCE data, with information on sociodemographic, smoking, and alcohol history. Associations were assessed using logistic regression with 95% confidence intervals (CI). The study included 2143 cases with HNC and 5799 controls. Cancer cases were categorized by their respective subsites: 954 LC (44.5%), 685 OPC (32.0%), 504 OCC (23.5%). The risk of developing OCC was 2.8‐fold higher [CI: 1.9–4.1], LC 2.6‐fold higher [CI: 1.9–3.5], and OPC 2.1‐fold higher [CI: 1.5–2.8] in individuals who smoked > 50 pack‐years, compared to < 10 pack‐years. The risk of OCC/OPC/LC increased with tobacco consumption in North‐America, whereas in Western/Southern‐Europe and South‐America the association plateaued beyond 31–50 pack‐years. Cessation after age 55 increased the risk of LC by 3.0‐fold [CI: 2.2–4.2], and OCC by 2.2‐fold [CI: 1.4–3.3] versus cessation age ≤ 45 years. Consuming ≥ 5 drinks/day was associated with 5‐fold higher risk of OPC [CI: 3.7–6.6], 4.4‐fold higher risk of OCC [CI: 3.2–6.1] and 3.1‐fold higher risk of LC [CI: 2.4–3.9] compared to 0–0.9 drinks/day. The risk of HNC among former smokers is not homogeneous across regions and that there were distinct patterns for OCC, OPC, and LC. The amount of tobacco and alcohol consumption are key risk factors, with alcohol being more important for OCC/OPC, and tobacco being more strongly associated with LC risk.

## Abstract (highlights)

What's new?

Tobacco use and alcohol consumption are primary risk factors for head and neck squamous cell carcinoma, with combined use occurring in three‐quarters of cases. This large international study of former smokers shows that the risk reduction effect of smoking cessation occurs more slowly for laryngeal cancer than for other head and neck cancer subsites. The persistent elevated risk highlights the need for long‐term surveillance of former smokers. The strong tobacco–alcohol synergy also calls for integrated interventions encouraging concurrent cessation. Public health policies should prioritize early prevention and adopt region‐specific strategies to reduce the global head and neck cancer burden.

## Graphical abstract

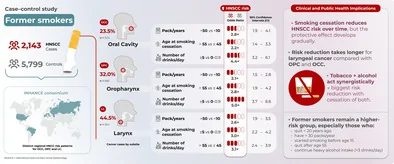

**Abbreviations**
95% CI: 95% confidence intervals
CI: confidence intervals
HNC: Head and neck cancer
HNSCC: Head and neck squamous cell carcinoma
HPV 16: Human papillomavirus
ICD‐O‐3: International Classification of Diseases for Oncology
INHANCE: International Head and Neck Cancer Epidemiology Consortium
LC: Laryngeal cancer
OCC: Oral cavity cancer
OPC: Oropharyngeal cancer
OR: Odds ratios

## 1 Introduction

In 2022, head and neck cancer (HNC, comprising cancers of the oral cavity, oropharynx, hypopharynx, and larynx) was the seventh‐most‐common malignancy worldwide, accounting for 771,000 new cases and 384,000 deaths. Projections indicate these cancers will increase approximately 30% by 2030 [1]. Oral cavity cancer (OCC) was the most common in 2022, with 389,496 cases, followed by laryngeal cancer (LC) with 189,191 cases, and oropharyngeal cancer (OPC) with 106,400 cases [1].

The primary risk factors for head and neck squamous cell carcinoma (HNSCC) are tobacco use and alcohol consumption [2], with combined use occurring in 72% of cases [2, 3, 4]. Smoking poses the greatest risk for LC, while alcohol consumption is more strongly associated with OCC and OPC [5]. Another risk factor is infection with human papillomavirus (HPV), with the proportion of OPC cases attributed to viral infection ranging from 17% to 56% in high income countries and 13% in low‐ and middle‐income countries [6, 7, 8].

There is a strong association between the intensity and duration of smoking and HNSCC risk [2, 9]. However, smokers can lower their subsequent risk of these cancers by smoking cessation [9, 10, 11, 12]. A recent meta‐analysis showed a reduction in the risk of HNC within the first 5 years after quitting smoking, with similar effects across oral cavity, laryngeal, and oropharyngeal cancer [13]. Previous evidence also supported an inverse association between smoking cessation and HNSCC risk [11, 12, 14, 15]. However, alcohol consumption in former smokers has not been comprehensively analyzed by HNSCC subsite [10].

The risk of developing specific subsites of HNSCC among former smokers is less well known; as most studies have focused on the risk by years since quitting. The aim of this study, based on a large international consortium, was to identify differences in HNSCC risk among former smokers by subsite (oral cavity, oropharynx, and larynx) and to evaluate the impact of age, duration, and amount of tobacco and alcohol consumption.

## 2 Material and Methods

The data were obtained from the INHANCE Consortium (https://medicine.utah.edu/dfpm/inhance), including data collected during 1984–2013. The study included former smokers with squamous cell carcinoma (Morphological Code 8070/3) of the oral cavity, oropharynx, and larynx, according to the International Classification of Diseases for Oncology (ICD‐O‐3, 2000): oral cavity (C00.3; C00.4; C00.5; C00.6; C00.7; C00.8; C00.9; C02.0; C02.1; C02.2; C02.3; C02.4; C02.8; C02.9; C03.0; C03.1; C03.9; C04.0; C04.1; C04.8; C04.9; C05.2; C05.8; C05.9; C06.0; C06.1; C06.2; C06.8; C06.9), oropharynx (C01.9; C05.1; C09.0; C09.1; C09.8; C09.9; C10.0; C10.2; C10.3; C10.4; C10.8; C10.9) and larynx (C10.1; C32.0; C32.1; C32.2; C32.3; C32.8; C32.9), and controls. Hypopharyngeal cancer was not included because the number of former smoker cases was fewer than 300. In the INHANCE dataset, former smokers were defined as individuals who had stopped smoking for more than 1 year.

The INHANCE database included 63,113 participants, comprising 25,865 HNSCC cases and 37,248 controls. The pooled analysis included 16 studies from the database with available individual‐level data on smoking cessation and all covariates. These studies represent the subset of INHANCE studies that provided data for the present analysis. The total number of cases in these studies was 11,114, and the total number of controls was 17,155. These studies were conducted in the USA (6), Italy (5), Germany (2), Brazil (1), France (1), Central Europe (1). Study designs comprised both hospital‐based and population‐based studies. For the population‐based studies, the case sources were cancer registries from the respective countries. Nine studies were matched by at least sex and age (Table 1).

**TABLE 1:** Characteristics of study participants included in the pooled analysis (INHANCE Consortium; 1984–2013).

Study name	Study location	Institute	Period of recruitment	Case‐control	Case source	Case participatio*n*, %	Control source	Control participation, %	Matched factores	Cases, *n* (%)	Controls, *n* (%)	Total, *n* (%)
Milan (1984–1989)	Milan, Italy	Mario Negri	1984–1989	Hospital‐based	Hospital	95	Hospital—unhealthy	95	—	416 (3.7)	1531 (8.9)	1947 (6.9)
Aviano	Aviano, Italy	CRO	1987–1992	Hospital‐based	Hospital	> 95	Hospital—unhealthy	95	—	482 (4.3)	855 (5.0)	1337 (4.7)
Italy Multicenter	Italy	Italy Multicenter	1990–1999	Hospital‐based	Hospital	> 95	Hospital—unhealthy	95	—	1261 (11.3)	2716 (15.8)	3977 (14.1)
Central Europe	Central Europe	IARC	1998–2003	Hospital‐based	Hospital	96	Hospital—unhealthy	97	Age, sex, ethnicity, city	762 (6.9)	907 (5.3)	1669 (5.9)
Seattle (1985–1995)	Seattle, WA, USA	FHCRC		Population‐based						407 (3.7)	607 (3.5)	1014 (3.6)
Los Angeles	Los Angeles, CA, USA	UCLA	1999–2004	Population‐based	Cancer registry	49	Neighborhood	68	Age, sex, neighborhood	417 (3.8)	1005 (5.9)	1422 (5.0)
Boston	Boston, MA, USA	Harvard	1999–2003	Population‐based	Hospital	88.7	Residential records	48.7	Age, sex, neighborhood	584 (5.3)	659 (3.8)	1243 (4.4)
Rome	Rome, Italy	Università Cattolica del Scaro Cuore	2002–2007	Hospital‐based	Hospital	98	Hospital—unhealthy	94	—	361 (3.2)	396 (2.3)	757 (2.7)
Sao Paulo	Sao Paulo, Brazil	Sao Paulo University	2002–2007	Hospital‐based	Hospital		Hospital—unhealthy			1922 (17.3)	1670 (9.7)	3592 (12.7)
Germany‐Saarland	Germany	DKFZ	2001–2003	Population‐based	Hospital	94	Health examination		Age, sex	94 (0.8)	94 (0.5)	188 (0.7)
Germany‐Heidelberg	Germany	University of Heidelberg	1998–2000	Population‐based	Hospital	96	Population registries	62.4	Age, sex, residence	228 (2.1)	769 (4.5)	997 (3.5)
North Carolina (2002–2006)	North Carolina, USA	UNC (Population based)	2002–2006	Population‐based	Cancer registry	82	Department of Motor/Vehicles files	61	Age, sex, ethnicity	1368 (12.3)	1396 (8.1)	2764 (9.8)
HOTSPOT	Baltimore, Boston, Portland	Johns Hopkins	2009–2013	Hospital‐based	Hospital	> 85	Hospital—benign conditions	> 80	Age, sex, ethnicity	71 (0.6)	71 (0.4)	142 (0.5)
France Multicenter (2001–2007)	Paris, France	INSERM	2001–2007	Population‐based	Cancer registry	82.5	Random digit dialing	80.6	Age, sex, region	2237 (20.1)	3555 (20.7)	5792 (20.5)
Milan (2006–2009)	Milan, Italy	Mario Negri	2006–2009	Hospital‐based	Hospital	> 95	Hospital	> 95	—	370 (3.3)	755 (4.4)	1125 (4.0)
MSKCC	New York, USA	MSKCC	1992–1994	Hospital‐based	Hospital	—	Blood donors	—	Age, sex	134 (1.2)	169 (1.0)	303 (1.1)

The variables collected included: sex at birth (male and female), age (stratified into < 60, 60–69, and ≥ 70 years), ethnicity (White, Black, Brazilian, and others), educational level (0–8, 9–12, > 12 years), and annual household income (≤ US$29,999; US$30,000–59,999; ≥ US$60,000) and family history of tobacco‐related cancer (yes or no). For ethnicity classification, the “others” category included Asians, Pacific Islanders and Hispanics. Smoking variables included pack‐years (0.1–20; 21–40; > 40), age at smoking initiation (< 15; 16–20; > 21 years), age at smoking cessation (< 45; 46–55; > 55 years), and years since smoking cessation (1–10; 11–20; 21–30; > 30 years). Alcohol consumption variables included drinking status (never drinker; former drinker; current drinker), number of drinks per day (1 drink equivalent to 15.6 mL of alcohol, stratified as never drinker; < 1; 1–2.9; 3–4.9; > 4.9), age at alcohol initiation (< 15; 16–20; > 20 years), age at alcohol cessation (< 45; 46–55; > 55 years), and years since alcohol cessation (1–10; 11–20; > 20 years).

The chi‐square test was used for categorical variables, with a *p*‐value < 0.05 considered statistically significant. Odds ratios (OR) with corresponding 95% confidence intervals (95% CI) were calculated using logistic regression analysis. Multiple (multivariable) logistic regression was performed for variables that were statistically significant in the univariate model (*p* < 0.20). In the multiple logistic regression analysis, only variables with less than 5% of missing values were considered. Missing values were grouped into a separate category. The final model was defined based on the following criteria: (1) no change in OR > 10%; (2) statistical significance of variables within the multiple model (*p* < 0.05); and (3) the total degrees of freedom allowed for the outcome. The Hosmer‐Lemeshow test was applied to assess model fit. Three models were created to identify subsite‐specific risk associations among former smokers. Due to study heterogeneity, a random effect for the study variable was included in all three regression models. For subsite comparisons, the variables included in the final model did not differ. The models adjusted for age, sex, and ethnicity can be found in Table S2. Additionally, regression models per subsite were created based on the variables with the strongest associations in previous analyses to assess differences in risk associations among former smokers across geographic locations (as defined by the studies). All statistical analyses were performed using the R programming language within the RStudio environment.

## 3 Results

This pooled analysis included 16 studies from the INHANCE Consortium, comprising 11,114 cases, and 17,155 controls. Among these, 7515 individuals were former smokers, of whom 2143 were HNSCC cases and 5372 were controls without HNSCC (Table 2). In the pooled dataset, studies from Italy accounted for 31.0% of the sample, followed by the USA (21.9%), France (19.6%), and Brazil (12.2%) (Table 1). The distribution of cases by subsite was as follows: 954 laryngeal cancers (44.5%), 685 oropharyngeal cancers (32.0%), and 504 oral cavity cancers (23.5%).

**TABLE 2:** Sociodemographic characteristics of 2143 former smokers with HNSCC by subsite and 5372 controls (INHANCE Consortium; 1984–2013).

Characteristics	Controls	Oral cavity	Oropharynx	Larynx	*p*
	*n* (%)	*n* (%)	*n* (%)	*n* (%)	
Sex					< 0.001
Male	4600 (85.7)	421 (84.2)	603 (88.0)	900 (94.3)	
Female	765 (14.3)	79 (15.8)	82 (12.0)	54 (5.7)	
Missing	7	4	0	0	
Age (years)					< 0.001
< 60	2570 (47.8)	218 (43.3)	378 (55.2)	305 (32)	
60–69	1758 (32.7)	186 (36.9)	221 (32.3)	385 (40.4)	
≥ 70	1044 (19.4)	100 (19.8)	86 (12.6)	264 (27.7)	
Missing	0	0	0	0	
Ethnicity					< 0.001
White	4604 (85.7)	338 (67.1)	592 (86.4)	756 (79.2)	
Black	155 (2.9)	16 (3.2)	29 (4.2)	47 (4.9)	
Brazilian	489 (9.1)	146 (29)	53 (7.7)	144 (15.1)	
Others	124 (2.3)	4 (0.8)	11 (1.6)	7 (0.7)	
Missing	17	0	0	1	
Education (years)					< 0.001
0–8	1791 (33.7)	204 (42.4)	220 (33.8)	412 (44.7)	
9–12	1747 (32.9)	170 (35.3)	241 (37.1)	332 (36)	
≥ 12	1774 (33.4)	107 (22.2)	189 (29.1)	178 (19.3)	
Missing	60	23	35	32	
Income (US$)					< 0.001
≤ US$29,999	284 (32.6)	33 (56.9)	63 (35.6)	105 (56.8)	
US$30,000–US$59,999	223 (25.6)	15 (25.9)	43 (24.3)	39 (21.1)	
≥ US$60,000	364 (41.8)	10 (17.2)	71 (40.1)	41 (22.2)	
Missing	4501	446	508	769	
Family history of cancer*					0.002
Yes	718 (19.7)	71 (26.2)	103 (24.2)	142 (24.2)	
No	2928 (80.3)	200 (73.8)	322 (75.8)	445 (75.8)	
Missing	1726	233	260	367	

* Tobacco‐related cancer. Missing values are not part of the chi‐square tests.

### 3.1 Sociodemographic Characteristics of Former Smokers

Male sex accounted for 94.3% (*n* = 900) of LC cases, 88.0% (*n* = 603) of OPC cases, and 84.2% (*n* = 421) of OCC cases (*p* < 0.001) (Table 2). Participants aged < 60 years represented 55.2% of OPC cases, while those aged ≥ 70 years accounted for 27.7% of LC cases (*p* < 0.001). Regarding educational level, 44.7% of LC patients had attained a low educational level (< 9 years of education) compared to 33.7% of controls (*p* < 0.001). Patients with HNC had a higher frequency of family history of tobacco‐related cancers compared with controls, ranging from 24.2% (OPC and LC) to 26.2% (OCC), compared to controls (19.7%) (*p* = 0.002). Data on family history of tobacco‐related cancers were missing for approximately one third of patients.

### 3.2 Tobacco Use in Former Smokers

In more than half of the cases (> 50%), participants had quit smoking within the past 10 years, 59.7% of OCC, 55.5% of OPC, and 54.6% of LC cases had stopped within this time window (*p* < 0.001) (Table 3). The most frequent tobacco consumption was 11–50 pack‐years across all subsites for cases (LC 60.7%, OCC and OPC 54.9%) and 0.1–30 pack‐years (71.1%) for controls (*p* = 0.001). Approximately 87%–89% of OCC, OPC, and LC cases compared to 84% of controls started smoking before the age of 20 years (*p* = 0.001). Smoking cessation before the age of 45 years was more common among OPC (42.0%) and OCC (37.9%) cases, whereas cessation after the age of 55 years was more frequent in LC patients (38.1%) (*p* < 0.001).

**TABLE 3:** Tobacco and alcohol consumption of 2143 former smokers' cases with HNSCC by subsite and 5372 controls (INHANCE Consortium; 1984–2013).

Characteristics	Controls	Oral Cavity	Oropharynx	Larynx	*p*
	*n* (%)	*n* (%)	*n* (%)	*n* (%)	
Tobacco (pack‐years)					< 0.001
0.1–10	1829 (34.6)	66 (13.5)	126 (18.6)	92 (9.9)	
11–30	1931 (36.5)	135 (27.6)	190 (28.1)	294 (31.7)	
31–50	852 (16.1)	134 (27.3)	181 (26.8)	269 (29.0)	
> 50	677 (12.8)	155 (31.6)	179 (26.5)	272 (29.3)	
Missing	83	14	9	27	
Age at smoking initiation (years)					< 0.001
≤ 15	2312 (43.3)	261 (52.5)	375 (54.9)	456 (48.3)	
16–20	2178 (40.8)	174 (35.0)	235 (34.4)	366 (38.7)	
> 20	850 (15.9)	62 (12.5)	73 (10.7)	123 (13.0)	
Missing	32	7	2	9	
Age at smoking cessation (years)					< 0.001
≤ 45	3411 (63.6)	191 (37.9)	288 (42.0)	292 (30.8)	
46–55	1167 (21.8)	143 (28.4)	223 (32.6)	295 (31.1)	
> 55	787 (14.7)	170 (33.7)	174 (25.4)	361 (38.1)	
Missing	7	0	0	6	
Time since smoking cessation (years)					< 0.001
1–10	1751 (32.7)	301 (59.7)	380 (55.5)	518 (54.6)	
11–20	1453 (27.1)	106 (21.0)	146 (21.3)	223 (23.5)	
21–30	1202 (22.5)	46 (9.1)	93 (13.6)	118 (12.4)	
> 30	947 (17.7)	51 (10.1)	66 (9.6)	90 (9.5)	
Missing	19	0	0	5	
Drinking status					< 0.001
Never	450 (10.9)	43 (10.2)	32 (5.2)	42 (6.1)	
Former	563 (13.7)	143 (33.8)	203 (33.1)	190 (27.5)	
Current	3106 (75.4)	237 (56.0)	379 (61.7)	460 (66.5)	
Missing	1253	81	71	262	
Number of drinks per day					< 0.001
0–0.9 drinks	2270 (43.1)	136 (28.3)	166 (24.8)	229 (25.1)	
1–2.9 drinks	1513 (28.7)	108 (22.5)	182 (27.2)	207 (22.6)	
3–4.9 drinks	717 (13.6)	72 (15.0)	106 (15.8)	157 (17.2)	
≥ 5 drinks	764 (14.5)	165 (34.3)	215 (32.1)	321 (35.1)	
Missing	108	23	16	40	
Age at drinking initiation (years)					< 0.001
Never drinker	512	46	33	58	
≤ 15	2097 (43.4)	236 (52.3)	362 (55.7)	432 (48.6)	
16–20	1977 (41.3)	161 (35.7)	224 (34.5)	346 (39.0)	
> 20	761 (15.3)	54 (12.0)	64 (9.8)	110 (12.4)	
Missing	25	7	2	8	
Age at drinking cessation (years)					< 0.001
Never drinker	519	45	38	61	
≤ 45	349 (35.8)	52 (28.4)	82 (34.2)	56 (28.4)	
46–55	313 (32.2)	62 (33.9)	85 (35.4)	55 (27.9)	
> 55	312 (32.0)	69 (37.7)	73 (30.4)	86 (43.7)	
Current drinker	2688	195	344 (55.3)	448 (63.5)	
Missing	1191	81	63	248	
Time since drinking cessation (years)					< 0.001
Never drinker	362	39	25	52	
1–10	204 (49.0)	92 (67.2)	100 (68.5)	105 (64.4)	
11–20	115 (27.6)	32 (23.6)	31 (21.2)	35 (21.5)	
> 20	97 (23.4)	13 (8.2)	15 (10.3)	23 (14.1)	
Current drinker	1999	158	285	320	
Missing	2595	170	229	419	

*Note:* Missing values are not part of the chi‐square tests.

**FIGURE 1:**
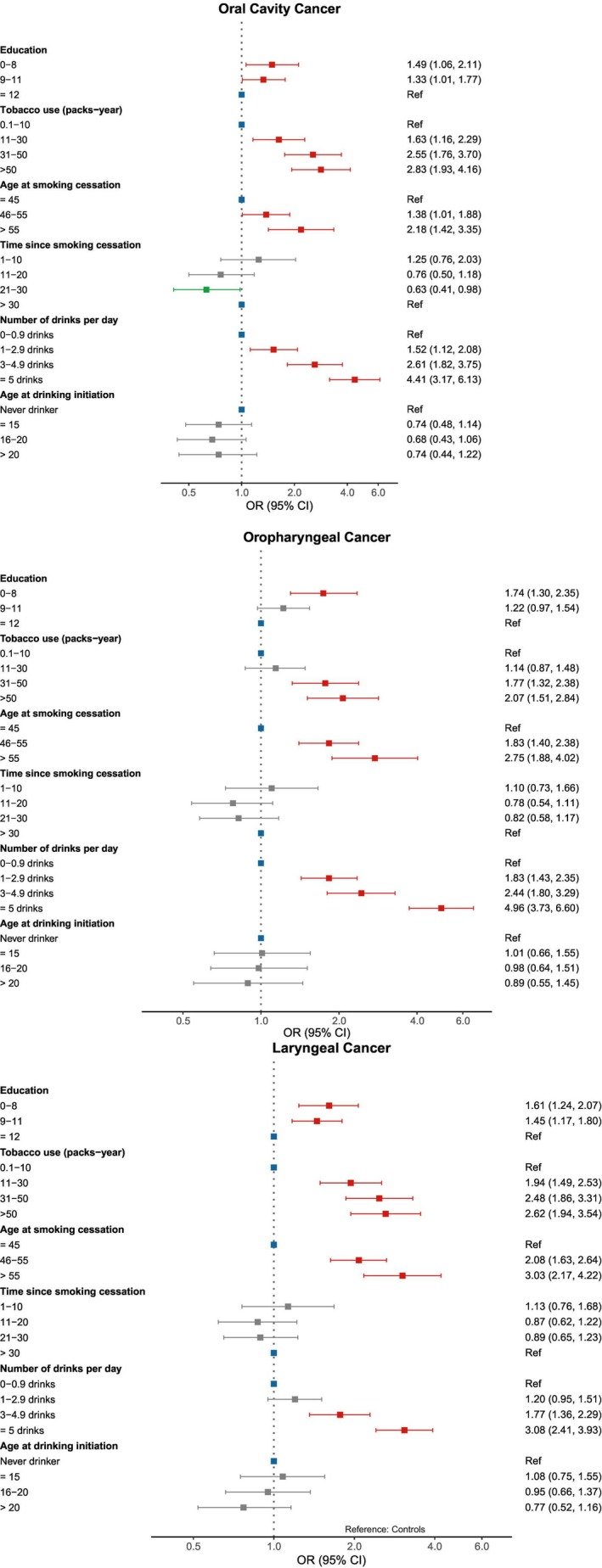
Adjusted logistic regression analysis (multiple models) of 2143 former smokers with OCC, OPC, and LC compared with controls (INHANCE Consortium, 1984–2013). Red dots indicate increased risk; green dots indicate decreased risk; gray dots indicate nonsignificant results; blue dots represent the reference category. Models were adjusted for sex, age, ethnicity, and study center (random effect).

Former smokers who smoked the highest number of pack‐years (> 50) had 2.8 times higher odds of developing OCC [CI: 1.9–4.2], 2.6 times higher odds of LC [CI: 1.9–3.5] and 2.1 times higher odds of OPC [CI: 1.5–2.8] compared with those who smoked ≤ 10 pack‐years (Figure 1; Table S2). The association of age at cessation of tobacco consumption with cancer risk was stronger for LC (3.0 times [CI: 2.2–4.2] for those who quit smoking after age 55 compared with cessation of tobacco consumption at age ≤ 45 years), followed by OPC (2.7 times [CI: 1.9–4.0]), and OCC (2.2 times [CI: 1.4–3.3]). Smoking initiation before the age of 15 years was associated with an increased the probability of developing OPC (1.71 times [CI: 1.3–2.2]), OCC (1.40 times [CI: 1.1–1.9]), and LC (1.32 times [CI: 1.1–1.6]), compared with starting smoking after age 20 years. Compared with a smoking cessation period of more than 30 years, a cessation period of 1–10 years was associated with a 3.2‐fold higher risk of LC [CI: 2.5–4.0], a 3.0‐fold higher risk of OCC [CI: 2.2–4.1], and a 2.9‐fold higher risk of OPC [CI: 2.2–3.8] (Table S2). However, this association disappeared after adjustment for other variables (Figure 1; Table S2).

### 3.3 Alcohol Consumption in Former Smokers

Most controls were current drinkers (*n* = 3106, 75.4%), among cases the highest proportion of current drinkers were seen among LC cases (66.5%), followed by OPC (61.7%) and OCC (56.0%) cases (*p* < 0.001) (Table 3). Notably, more than 20% of data on drinking status were missing. Drinking cessation after the age of 55 was reported by 43.7% (*n* = 86) of LC cases, 37.7% (*n* = 69) of OCC cases, and 30.4% (*n* = 73) of OPC cases, whereas the highest proportion of controls stopped drinking at age ≤ 45 years (35.8%, *n* = 349) (*p* < 0.001). Among those who had quit alcohol consumption, over 60% of individuals in the case group (OPC: 68.5%; OCC: 67.2%; LC: 64.4%) had quit alcohol consumption within the last 10 years, versus 49% among controls (*p* < 0.001). Data on time since drinking cessation were missing for approximately 45% of patients.

Former drinkers were 5.1 times more likely to develop OPC than never drinkers [CI: 3.5–7.6], 3.9 times more likely to develop LC [CI: 2.8–5.7] and 2.6 times more likely to develop OCC [CI: 1.9–3.9] (Table S1). Consuming ≥ 5 drinks per day was associated with more than 5 times higher risk of OPC [CI: 3.7–6.6], 4.5 times higher risk of OCC [CI: 3.3–6.1] and 3.1 times higher risk of LC [CI: 2.4–3.9] compared to the lowest alcohol consumption (0–0.9 drinks per day) (Figure 1, Table S2). Former smokers who stopped alcohol drinking after the age of 55 had a 3.7 times higher risk of developing LC than never drinkers [CI: 2.5–5.3], 3.1 times higher risk of OPC [CI: 2,1–4.8], and 2.5 times higher risk of OCC [CI: 1.7–3.8] (Table S1). A shorter time period since alcohol cessation of 1–10 years was associated with a 7.0 times higher risk of OPC [CI: 4.5–11.5] compared with never drinkers, 4.2 times higher risk of OCC [CI: 2.8–6.4], and 4.0 times higher risk of LC [CI: 2.8–5.9].

### 3.4 Geographic Location and Head and Neck Cancer Risk in Former Smokers

**FIGURE 2:**
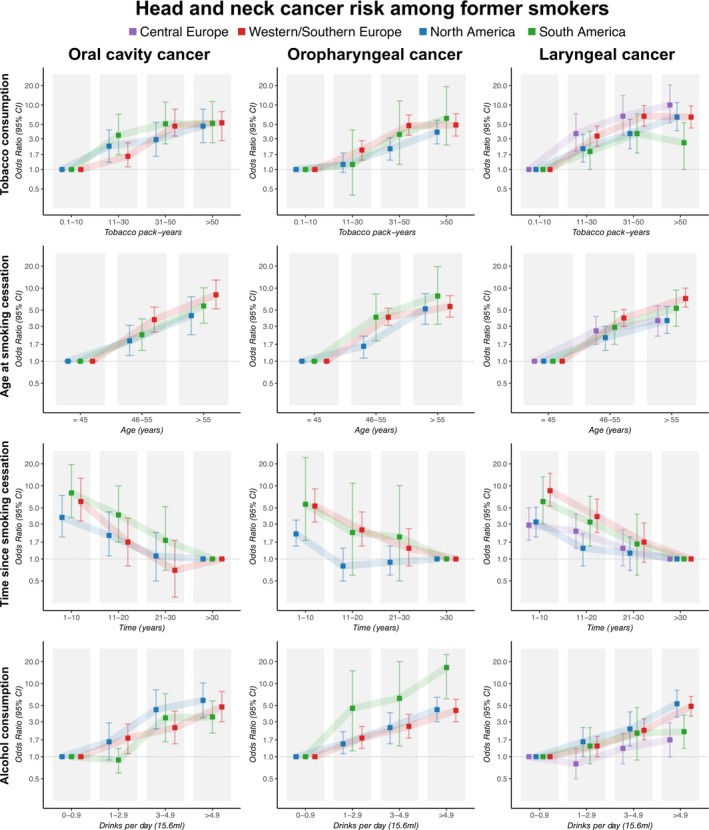
Association between oral cavity, oropharyngeal, and laryngeal cancer among former smokers by geographic location (INHANCE Consortium; 1984 to 2013). Central Europe corresponding to Slovakia, Romania, Hungary, Poland, Germany, and Russia. Western/Southern Europe corresponding to Italy and France. All models were adjusted for sex and age.

Among all regions, the likelihood of developing OCC, OPC, and LC increased with the number of pack‐years; however, the association plateaued in some regions but not in others. Among former smokers in North America, the likelihood of developing OCC, OPC, and LC increased with the number of pack‐years (OR 5.9 for OCC; 3.8 for OPC; and 3.6 for LC for those with > 50 pack‐years compared with the 0.1–10 pack‐years group) (Figure 2; Table S3). In Western/Southern Europe and South America, the association plateaued from 31 to 50 pack‐years onwards, except for OPC in South America, where the risk continued to increase (6.2 times more likely for > 50 pack‐years compared with 0.1–10 pack‐years). Those who quit smoking after the age of 55 were more likely to develop OCC, OPC, and LC compared with those who quit before the age of 45, with the association being more pronounced in South America (5.3‐fold higher risk of LC) and Western/Southern Europe (7.2‐fold higher risk of LC), compared with North America and Central Europe (3.6‐fold higher risk of LC in both regions).

For OCC, equivalent risk levels similar to those who had quit for > 30 years were observed only in the 21–30 years since cessation group in South and North America, whereas in Western Europe it occurred earlier, at 11–20 years (OR: 1.7 [CI: 0.8–3.6]) (Figure 2; Table S3). For OPC, Western Europe equivalence occurred only after 21–30 years, while the 11–20 years group remained at higher risk (OR: 2.5 [CI: 1.5–4.4]) compared to those who quit > 30 years ago, while in North (OR: 0.8 [CI: 0.6–1.5]) and South America (OR: 2.3 [CI: 0.6–10.9]) no differences were observed from 11 years since cessation. Former smokers in South America, Central Europe, and Western/Southern Europe remained more likely to develop LC up to 20 years after cessation. In contrast, in North America the excess risk did not persist after 11 years since cessation, when compared with those who had quit for more than 30 years (OR: 1.4 [CI: 0.8–2.5]).

Alcohol consumption above 3 drinks per day, compared with 0–0.9 drinks, was associated with higher odds of OCC, OPC, and LC in all regions (Figure 2; Table S3). Except for OPC, this association was stronger among former smokers in North America (5.3 times more likely for LC and 5.9 times more likely for OCC among those consuming > 4.9 drinks per day).

## 4 Discussion

The risk reduction effect of smoking cessation occurs more slowly for LC compared with other HNSCC subsites. Approximately 56% of former smokers had quit smoking within 10 years, and this shorter time interval was positively associated with the risk of HNSCC across all subsites in all geographic locations compared with those who had quit for > 30 years. The highest odds of HNSCC among former smokers were found among individuals with low educational attainment, particularly those with OCC and LC. Conway et al. observed a 2.5‐fold higher risk of HNSCC among individuals with lower education and income [16]. In the present study, there was an association between lower education and income with OCC, OPC, and LC risk among former smokers, although a higher proportion of individuals with higher education was found among former smokers with OPC compared with other HNSCC.

Tobacco consumption of > 30 pack‐years (vs. 0.1–10 pack‐years) was associated with an increased risk of HNSCC in all subsites, corroborating previous studies [9, 11, 17]. For LC, the risk increased by 3‐fold for former smokers with tobacco use of 40 cigarettes per day after 10 years of cessation [18]. The current results showed a 2.6‐fold greater risk for LC among former smokers with the highest tobacco consumption (> 50 pack‐years). In Spain, former smokers who consumed more than 20 cigarettes per day had a 30.8‐fold higher risk of developing OPC/OCC compared with those who consumed 20 cigarettes per day or less, according to Varela‐Lema et al. [11]. In the present study, when analyzing OCC and OPC separately, we found different risks: a 2.5‐fold increase in OCC risk and a 1.8‐fold increase in OPC risk among those who consumed 31–50 pack‐years, compared with those who consumed 0.1–10 pack‐years. Di Credico et al. noted that the dose–response relationship for OPC, OCC, and LC was nonlinear, with a statistically significant increase for intermediate tobacco consumption (20–30 cigarettes per day), followed by a “saturation effect” among individuals with a smoking history of > 20 years and consumption of > 30 cigarettes per day [9]. This result is consistent with the present analysis of former smokers, where no further statistically significant increase in risk for OPC, OCC, or LC was observed at consumption levels exceeding 30 pack‐years, both overall and within specific geographic regions.

Regarding age at tobacco initiation, former smokers with HNSCC across all subsites began smoking before the age of 20 years, with the majority starting before age 15. In Spain, men who started smoking after age 15 had a 50% lower risk of oral and oropharyngeal cancer [11]. The current study data showed that those who started smoking before age 15 had the highest risk increase (70%) for OPC, while the risk was lower for the other subsites (< 40%), demonstrating that the earlier smoking initiation occurs, the higher the risk of developing HNSCC. Former smokers who quit smoking before age 35 had an 86% lower risk for OPC/OCC and 89% lower risk for LC compared with quitting after age 65, according to Bosetti et al. [14]. In the present study, individuals who quit smoking after age 55 years had 3.0 times greater risk of developing LC, 2.7 times greater for OPC, and 2.2 times greater for OCC compared to those who stopped before 45 years of age. These findings are consistent with the current literature, showing that younger former smokers exhibit a lower risk of developing HNSCC [14].

More than half of former smokers in this study had stopped smoking within the last 10 years, and this shorter interval since cessation, compared with > 30 years since cessation, was associated with an elevated risk of HNSCC across all subsites in all geographic regions (America and Europe). Bosetti et al. found that former smokers had an 88% and 82% lower risk of OPC/OCC and LC, respectively [14]. Former smokers who had quit smoking < 10 years prior, compared with > 30 years prior, exhibited a 4.6 times greater risk of developing LC. This risk can be as much as one third lower for LC and by half for OPC/OCC compared to current smokers [19]. In the current investigation, when analyzing all cases, no increased risk of OCC, OPC, or LC was observed among former smokers who had quit 1–10 years prior compared with those who had quit > 30 years earlier, after controlling for other variables such as tobacco and alcohol consumption. This suggests that the association between time since cessation and HNSCC risk should be interpreted cautiously, as it may be attenuated after adjustment for other tobacco‐related risk factors. Furthermore, the comparison between former smoker cases and controls failed to show the linear reduction in OPC and LC risk, which was previously described by Schlecht [20]. The literature remains inconsistent regarding the timeline for HNSCC risk reduction in former smokers. Some authors report that the risk across all subsites begins to decline within the first few years after quitting tobacco smoking, while larger risk reductions can be observed 15–20 years after cessation [11, 14, 15, 19, 21].

Strong interactions exist between alcohol consumption and tobacco use in individuals with HNSCC [2, 11]. Previous studies have shown that alcohol is a significant modifier of the association between OCC and OPC risk with tobacco consumption [2, 9, 17]. The present study data revealed an association between higher alcohol consumption among former smokers and the risk of developing OCC, OPC, and LC, with heavy drinkers (> 5 drinks per day vs. 0–0.9 drinks per day) showing an approximately 5.0‐fold higher risk for OPC and 4.4‐fold higher risk for OCC, whereas the risk increase for LC was lower (3.1‐fold). Varela‐Lema et al. identified a dose–response association between OPC and OCC among moderate and heavy drinkers who also used tobacco [11]. Although the risk of HNSCC was present across all subsites, this increase in risk differed by subsite and also by geographic region. While Central Europe showed the lowest risk increases, North America presented the highest risk increases for OCC and LC, whereas South America showed the highest risk increases for OPC.

There was a higher proportion of alcohol cessation within 10 years among individuals with HNSCC compared with controls. Former smokers who quit alcohol consumption within this time frame, compared with never drinkers, had a higher risk of HNSCC, mainly OPC (7.0 times greater risk). Marron et al. [15] found that individuals who had stopped drinking more than 20 years prior exhibited a 40% lower risk of HNSCC compared with current drinkers. In our study, participants who were both former smokers and former drinkers still presented increased risks for all HNSCC subsites, possibly because the risk conferred by alcohol consumption might be lower than that of tobacco [15]. Compared with tobacco cessation, a longer period is necessary to reduce HNSCC risk after alcohol cessation due to the lasting and irreversible effects associated with alcohol consumption [15, 22, 23, 24]. Marron et al. reported that quitting both smoking and drinking reduced the risk of HNSCC to that of never‐users after 20 years of stopping these habits [15]. These results are consistent with those of the present study on former smokers for just OCC, specifically, which showed the greatest extent of reduction after 20 years of quitting both smoking and drinking.

There are several strengths of this study. One of them is the large sample size of former smokers, including more than 2000 cases and 5000 controls from well‐designed multicentre case–control studies with harmonized data [24]. This sample provided adequate statistical power to detect associations among former smokers across head and neck cancer subsites. Although some variation in the magnitude of risk was observed across geographic regions, the results were overall consistent and demonstrated the robustness of the data across multiple studies. Another strength is that the availability of detailed information on tobacco and alcohol habits enabled the estimation of key risk factors and appropriate adjustment for potential confounders.

Our study has some limitations, including the lack of analysis of other factors, such as dietary habits and HPV infection. Although information on HPV infection was not available for this analysis, we observed consistent associations across all head and neck cancer subsites, regardless of their HPV‐related status. A further limitation concerns the analytical strategy, as unconditional logistic regression was used despite the presence of matched designs in some studies to allow pooling of studies with and without matching, with increased statistical power; models were adjusted for study center to account for variation between the different study designs. Variables such as income, drinking cessation, and family history of cancer had missing data and therefore could not be included in the association analyses. Another potential limitation includes the possibility of recall bias, as with any observational retrospective observational study. Misreporting of past behaviors, such as age at smoking initiation, duration and intensity of tobacco and alcohol use, may have occurred, leading to some degree of exposure misclassification. Since cases may recall their past behaviors more accurately or in greater detail than controls, this differential recall could have resulted in bias away from the null, potentially overestimating the true associations. Finally, differences in control selection and in geographic areas with varying recruitment periods may have introduced selection bias, depending on the underlying exposure distribution. Overall, these potential sources of bias may have acted in different directions, and their net impact on the estimates could not be determined.

### 4.1 Clinical and Public Health Implications

These findings have important implications for clinical practice and public health strategies. Healthcare providers should emphasize that while smoking cessation substantially reduces HNSCC risk over time, the protective effect develops gradually, with LC requiring a longer period to achieve risk reduction comparable to OPC and OCC. Clinicians should maintain heightened surveillance for HNSCC among former smokers, particularly those who: (1) quit smoking within the past 20 years, (2) have cumulative tobacco exposure exceeding 30 pack‐years, (3) initiated smoking before age 15, (4) quit smoking after age 55, and (5) continue heavy alcohol consumption (> 3 drinks per day). The synergistic effect of tobacco and alcohol emphasizes the critical importance of concurrent cessation of both substances to maximize risk reduction.

Prevention efforts should target early smoking initiation, as individuals who begin smoking before age 15 demonstrate significantly elevated risks across all HNSCC subsites. Public health campaigns must continue to discourage smoking initiation among adolescents and encourage early cessation, ideally before age 45, when the greatest risk reduction benefits are observed. Additionally, the geographic variation in risk patterns suggests that regional differences in tobacco products, consumption patterns, or genetic susceptibility may influence HNSCC risk, warranting tailored prevention strategies for different populations.

This large international study demonstrates that comprehensive tobacco control strategies must extend beyond promoting cessation to include long‐term risk management and surveillance of former smokers. The persistent elevated risk among those with high cumulative exposure and continued alcohol use highlights the need for integrated interventions addressing multiple behavioral risk factors to reduce the substantial burden of HNSCC worldwide.

### 4.2 Conclusion

In conclusion, the risk of HNSCC among former smokers is not homogeneous and shows distinct patterns for OCC, OPC, and LC. The risk reduction effect of smoking cessation occurs more slowly for LC compared with OPC and OCC. Nevertheless, total tobacco and alcohol consumption were stronger determinants of OCC, OPC, and LC risk among former smokers. Alcohol is a more significant risk modifier for OCC and OPC, whereas for LC, factors related to tobacco exposure are more strongly associated with the increased risk. Importantly, the persistence of elevated HNSCC risk among former smokers underscores that cessation alone does not immediately eliminate cancer risk, particularly within the first 10–20 years after quitting.

## Author Contributions

**Matheus de Abreu:** writing – original draft, formal analysis, data curation, investigation, methodology, validation, visualization, writing – review and editing, conceptualization, software. **Luiz Paulo Kowalski:** writing – review and editing, investigation, visualization, conceptualization, methodology. **Rossana Mendoza López:** conceptualization, investigation, methodology, formal analysis, validation, visualization, writing – review and editing, data curation, software. **Christine Barul:** writing – review and editing. **Loredana Radoi:** writing – review and editing. **Ettore Bidoli:** writing – review and editing. **Jerry Polesel:** writing – review and editing. **Victor Wunsch‐Filho:** writing – review and editing. **Andrew F. Olshan:** writing – review and editing. **Jose Zevallos:** writing – review and editing. **Eva Negri:** writing – review and editing. **Valeria Edefonti:** writing – review and editing. **Beata Świątkowska:** writing – review and editing. **Dana Mates:** writing – review and editing. **Eleonora Fabianova:** writing – review and editing. **Jolanta Lissowska:** writing – review and editing. **Oxana Shangina:** writing – review and editing. **Paul Brennan:** writing – review and editing. **Tamas Pandics:** writing – review and editing. **Luigino Dal Maso:** writing – review and editing. **Hal Morgenstern:** writing – review and editing. **Zuo‐Feng Zhang:** writing – review and editing. **Karl Kelsey:** writing – review and editing. **Michael McClean:** writing – review and editing. **Carlo La Vecchia:** writing – review and editing, methodology. **Werner Garavello:** writing – review and editing. **Chu Chen:** writing – review and editing. **Stephen M. Schwartz:** writing – review and editing. **Heribert Ramroth:** writing – review and editing. **Volker Winkler:** writing – review and editing. **Gabriella Cadoni:** writing – review and editing. **Stefania Boccia:** writing – review and editing. **Hermann Brenner:** writing – review and editing. **Gypsyamber D'Souza:** writing – review and editing. **Neil Gross:** writing – review and editing. **Joshua Muscat:** writing – review and editing. **Mahsa Abedini:** writing – review and editing. **Michele Sassano:** writing – review and editing. **Paolo Boffetta:** writing – review and editing, methodology. **Mia Hashibe:** methodology, writing – review and editing. **Yuan‐Chin Amy Lee:** methodology, writing – review and editing. **Maria Paula Curado:** conceptualization, investigation, writing – review and editing, visualization, validation, supervision, resources, methodology.

## Funding

The pooled data coordination team were supported by National Cancer Institute grant R03CA11315. This work was supported by funders of the original studies. The France 2001–2007 (ICARE): French National Research Agency (ANR); French National Cancer Institute (INCA); French Agency for Food, Environmental and Occupational Health and Safety (ANSES); French Institute for Public Health Surveillance (InVS); Fondation pour la Recherche Medicale (FRM); Fondation de France; Fondation ARC pour la Recherche sur le Cancer; French Ministry of Labour (Direction Generale du Travail); French Ministry of Health (Direction Generale de la Sante). The São Paulo study: Sao Paulo Research Foundation (FAPESP) (GENCAPO 04/12054‐9, 10/51168‐0). The North Carolina (2002–2006) study: NCI R01CA90731‐01 and NIEHS P30ES010126. The Milan study: Milan study: Italian Association for Research on Cancer (AIRC). The Aviano study: Italian Association for Research on Cancer (AIRC), Italian League Against Cancer and Italian Ministry of Research. The Central Europe study: World Cancer Research Fund and the European Commission INCO‐COPERNICUS Program [Contract No. IC15‐CT98‐0332]. The Los Angeles study: NIH (P50CA090388, R01DA011386, R03CA077954, T32CA009142, U01CA096134, R21ES011667) and the Alper Research Program for Environmental Genomics of the UCLA Jonsson Comprehensive Cancer Center. The Boston study: NIH (R01CA078609, R01CA100679). The Milan study (2006–2009) Italian Association for Research on Cancer (AIRC) and Italian Ministry of Education (PRIN 2009 X8YCBN). The Seattle study: NIH (R01CA048996, R01DE012609). The Germany‐Heidelberg study: grant No. 01GB9702/3 from the German Ministry of Education and Research. The Rome study: AIRC (Italian Agency for Research on Cancer). The MSKCC study: NIH [R01CA051845]. The Saarland study: Ministry of Science, Research and Arts Baden‐Wurttemberg. HOTSPOT: Johns Hopkins Richard Gelb Cancer Prevention Award. The NY multicenter study: NIH [P01CA068384 K07CA104231]. The author Matheus de Abreu received financial support from the Coordenação de Aperfeiçoamento de Pessoal de Nível Superior—Brasil (CAPES)—Finance Code 001, Process No. 88887.929140/2023‐00.

## Ethics Statement

Ethical approval was obtained from appropriate institutional local review boards.

## Consent

All participants provided written informed consent for the original studies.

## Conflicts of Interest

Neil D. Gross has potential financial conflicts of interest: Speaking Honoraria: AiCME, OncLive, Med Learning Group; Research Funding: Regeneron, Ascendis; Consulting: PDS Biotechnology, Pyxis, Merck, BMS, Trio Health, Regeneron, GeoVax. Royalties: Up‐to‐Date. Karl Kelsey has potential financial conflicts of interest: Founder and scientific advisor of Cellintec. These entities had no role in the design of the study, data collection, analysis, interpretation of data, or writing of the manuscript. All other authors declare no conflicts of interest.

## Supporting information


**Table S1:** Analysis of 2143 former smokers with HNSCC by subsite with logistic regression—simple models (INHANCE Consortium; 1984–2009).
**Table S2:** Analysis of 2143 former smokers with HNSCC by subsite with logistic regression—multiple models (INHANCE Consortium; 1984–2009).
**Table S3:** Analysis of 2143 former smokers with HNSCC by subsite and geographic location with logistic regression—multiple models (INHANCE Consortium; 1984–2009).

## Data Availability

All source code supporting the findings of this study is publicly available on GitHub at https://github.com/matheus‐deabreu/former‐smokers‐inhance.git. Users should access the repository and navigate to the “code” folder, which contains the script used for data analysis. Deidentified data and further information that support the findings of this study are available from the corresponding author upon reasonable request.
