# Supplementary material for: Risk of Head and Neck Cancer in Former Smokers by Subsite: A Multicenter Analysis From the INHANCE Consortium
Source: Int J Cancer. 2026 Apr 21;159(5):1204–17. doi: 10.1002/ijc.70497 (PMC13340975; doi:10.1002/ijc.70497)
Supplement: Supplementary file 1 — Table S1: Analysis of 2143 former smokers with HNSCC by subsite with logistic regression—simple models (INHANCE Consortium; 1984–2009). Table S2: Analysis of 2143 former smokers with HNSCC by subsite with logistic regression—multiple models (INHANCE Consortium; 1984–2009). Table S3: Analysis of 2143 former smokers with HNSCC by subsite and geographic location with logistic regression—multiple models (INHANCE Consortium; 1984–2009). [file IJC-159-1204-s001.pdf]

Risk of head and neck cancer in former smokers by subsite: a multicenter analysis from the INHANCE consortium

Authors: Matheus de Abreu, Luiz Paulo Kowalski, Rossana Mendoza López, Christine Barul, Loredana Radoi, Ettore Bidoli, Jerry Polesel, Victor Wunsch-Filho, Andrew F. Olshan, Jose Zevallos, Eva Negri, Valeria Edefonti, Beata Świątkowska, Dana Mates, Eleonora Fabianova, Jolanta Lissowska, Oxana Shangina, Paul Brennan, Tamas Pandics, Luigino Dal Maso, Hal Morgenstern, Zuo-Feng Zhang, Karl Kelsey, Michael McClean, Carlo La Vecchia, Werner Garavello, Chu Chen, Stephen M. Schwartz, Heribert Ramroth, Volker Winkler, Gabriella Cadoni, Stefania Boccia, , Hermann Brenner, Gypsyamber D'Souza, Neil Gross, Joshua Muscat, Mahsa Abedini, Michele Sassano, Paolo Boffetta, Mia Hashibe, Yuan-Chin Amy Lee, Maria Paula Curado

Table of contents

Supplementary Table 1 2

Supplementary Table 2 5

Supplementary Table 3 7

Supplementary Table 1. Analysis of 2,143 former smokers with HNSCC by subsite with logistic regression – simple models (INHANCE Consortium; 1984-2009).

	**Oral Cavity**			**Oropharynx**			**Larynx**		
**Variables**	**OR**	**CI (95%)**	**p**	**OR**	**CI (95%)**	**p**	**OR**	**CI (95%)**	**p**
**Sex**									
Male	0.93	0.73-1.21	0.607	1.29	1.02-1.66	**0.04**	2.71	2.06-3.65	**<0.001**
Female	Ref			Ref			Ref		
**Age**									
< 60	Ref			Ref			Ref		
60-69	1.27	1.03-1.56	**0.02**	0.87	0.72-1.04	**0.12**	1.84	1.56-2.16	**<0.001**
≥ 70	1.26	0.98-1.60	0.07	0.62	0.48-0.79	**<0.001**	2.00	1.67-2.39	**<0.001**
**Race**									
White	Ref			Ref			Ref		
Black	1.29	0.73-2.11	0.34	1.33	0.87-1.97	0.16	1.82	1.27-2.53	**<0.001**
Brazilian	3.73	3.00-4.61	**<0.001**	0.77	0.57-1.03	0.09	1.70	1.38-2.07	**<0.001**
Others	0.40	0.12-0.96	0.07	0.63	0.32-1.12	0.14	0.34	0.14-0.68	**<0.001**
**Education ***									
0-8	1.78	1.40-2.27	**<0.001**	1.08	0.88-1.33	0.43	2.05	1.70-2.48	**<0.001**
9-11	1.73	1.35-2.24	**<0.001**	1.39	1.14-1.71	**<0.001**	1.75	1.44-2.13	**<0.001**
≥ 12	Ref			Ref			Ref		
**Income**									
≤ US$29,999	4.23	2.12-9.18	**<0.001**	1.13	0.78-1.65	0.49	3.28	2.23-4.90	**<0.001**
US$30,000-US$59,999	2.45	1.09-5.71	**0.03**	0.98	0.65-1.49	0.95	1.55	0.97-2.48	0.06
≥ US$60,000	Ref			Ref			Ref		
**Family history of cancer***									
Yes	1.46	1.09-1.93	**0.01**	1.31	1.03-1.67	**0.02**	1.30	1.06-1.59	**0.01**
No	Ref			Ref			Ref		
**Tobacco use (packs-year)***									
0.1-10	Ref			Ref			Ref		
11-30	1.92	1.42-2.60	**<0.001**	1.41	1.20-1.79	**0.01**	3.01	2.37-3.86	**<0.001**
31-50	4.24	3.13-5.79	**<0.001**	3.00	2.36-3.83	**<0.001**	6.21	4.85-8.02	**<0.001**
>50	6.09	4.53-8.29	**<0.001**	3.69	2.89-4.72	**<0.001**	7.86	6.13-10.17	**<0.001**

**Age at smoking initiation***									
≤ 15	1.40	1.06-1.89	**0.02**	1.71	1.32-2.24	**<0.001**	1.32	1.07-1.64	**0.01**
16-20	1.08	0.80-1.48	0.60	1.24	0.95-1.65	0.12	1.18	0.95-1.48	0.13
> 20	Ref			Ref			Ref		
**Age at smoking cessation**									
≤ 45	Ref			Ref			Ref		
46-55	2.16	1.72-2.72	**<0.001**	2.24	1.86-2.71	**<0.001**	2.95	2.47-3.51	**<0.001**
> 55	3.88	3.10-4.84	**<0.001**	2.63	2.14-3.23	**<0.001**	5.17	4.35-6.15	**<0.001**
**Time since smoking cessation**									
1-10	2.98	2.21-4.10	**<0.001**	2.90	2.22-3.85	**<0.001**	3.16	2.50-4.03	**<0.001**
21-10	1.27	0.91-1.81	0.16	1.36	1.01-1.85	**0.05**	1.63	1.27-2.12	**<0.001**
21-30	0.70	0.46-1.05	0.08	1.09	0.79-1.52	0.60	1.04	0.79-1.39	0.77
> 30	Ref			Ref			Ref		
**Drinking status***									
Never drinker	Ref			Ref			Ref		
Former drinker	2.66	1.86-3.86	**<0.001**	5.07	3.47-7.63	**<0.001**	3.92	2.77-5.68	**<0.001**
Current drinker	0.80	0.67-1.45	0.19	1.71	1.20-2.54	**0.01**	1.68	1.22-2.38	**0.01**
**Number of drinks (15.6ml) per day***									
0-0.9 drinks	Ref			Ref			Ref		
1-2.9 drinks	1.25	0.96-1.63	0.08	1.73	1.39-2.16	**<0.001**	1.33	1.09-1.62	**<0.001**
3-4.9 drinks	1.82	1.34-2.45	**<0.001**	2.19	1.69-2.84	**<0.001**	2.06	1.65-2.57	**<0.001**
≥ 5 drinks	3.67	2.88-4.68	**<0.001**	3.92	3.15-4.88	**<0.001**	3.95	3.27-4.78	**<0.001**
**Age at drinking initiation***									
Never drinker	Ref			Ref			Ref		
≤ 15	1.27	0.92-1.79	0.16	2.71	1.90-4.00	**<0.001**	1.86	1.41-2.52	**<0.001**
16-20	1.01	0.72-1.43	0.96	1.96	1.36-2.91	**<0.001**	1.67	1.26-2.27	**<0.001**
> 20	0.89	0.59-1.34	0.58	1.47	0.96-2.30	0.08	1.36	0.97-1.91	0.07
**Age at drinking cessation***									
Never drinker	Ref			Ref			Ref		
≤ 45	1.69	1.11-2.59	**0.01**	3.16	2.12-4.80	**<0.001**	1.61	1.09-2.38	**0.01**
46-55	2.25	1.50-3.41	**<0.001**	3.66	2.45-5.55	**<0.001**	2.06	1.38-3.06	**<0.001**
> 55	2.52	1.69-3.78	**<0.001**	3.15	2.09-4.82	**<0.001**	3.66	2.54-5.29	**<0.001**
Current drinker	0.82	0.59-1.17	0.26	1.72	1.23-2.48	**0.01**	1.42	1.08-1.90	**0.01**

**Time since drinking cessation***									
Never drinker	Ref			Ref			Ref		
1-10	4.19	2.79-6.38	**<0.001**	7.10	4.50-11.58	**<0.001**	4.00	2.76-5.88	**<0.001**
11-20	2.58	1.54-4.31	**<0.001**	3.90	2.22-6.93	**<0.001**	2.50	1.53-4.05	**<0.001**
> 20	1.24	0.61-2.36	0.52	2.24	1.11-4.37	**0.02**	1.77	1.02-3.02	**0.04**
Current drinker	0.73	0.51-1.07	0.09	2.06	1.38-3.23	**<0.001**	1.10	0.81-1.65	0.55

*Missing were categorized as category for these variables.

Supplementary Table 2. Analysis of 2,143 former smokers with HNSCC by subsite with logistic regression – multiple models (INHANCE Consortium; 1984-2009).

	Oral Cavity[1], **			Oral Cavity[2], ***			Oropharynx[3], **			Oropharynx[4], ***			Larynx[5], **			Larynx[6], ***		
**Variables**	**OR**	**CI (95%)**	**p**	**OR**	**CI (95%)**	**p**	**OR**	**CI (95%)**	**p**	**OR**	**CI (95%)**	**p**	**OR**	**CI (95%)**	**p**	**OR**	**CI (95%)**	**p**
**Education ***																		
0-8	0.78	0.58-1.03	0.08	1.55	1.10-2.20	**0.01**	0.74	0.58-0.93	**0.01**	1.82	1.35-2.45	**<0.001**	1.12	0.91-1.40	0.28	1.71	1.32-2.21	**<0.001**
9-11	1.24	0.95-1.63	0.11	1.34	1.01-1.78	**0.04**	1.13	0.91-1.40	0.27	1.23	0.98-1.55	0.08	1.42	1.15-1.75	**0.001**	1.45	1.17-1.80	**<0.001**
≥ 12	Ref			Ref			Ref			Ref			Ref			Ref		
**Tobacco use (packs-year)***																		
0.1-10	Ref			Ref			Ref			Ref			Ref			Ref		
11-30	1.74	1.25-2.46	**0.001**	1.62	1.16-2.29	**0.01**	1.15	0.88-1.49	0.302	1.13	0.87-1.48	0.34	2.04	1.57-2.66	**<0.001**	1.93	1.49-2.52	**<0.001**
31-50	2.84	1.98-4.11	**<0.001**	2.54	1.76-3.70	**<0.001**	1.87	1.41-2.50	**<0.001**	1.77	1.32-2.37	**<0.001**	2.75	2.07-3.67	**<0.001**	2.45	1.84-3.28	**<0.001**
>50	3.47	2.40-5.05	**<0.001**	2.80	1.91-4.14	**<0.001**	2.18	1.62-2.95	**<0.001**	2.06	1.50-2.83	**<0.001**	3.22	2.42-4.33	**<0.001**	2.56	1.90-3.46	**<0.001**
**Age at smoking cessation***																		
≤ 45	Ref			Ref			Ref			Ref			Ref			Ref		
46-55	1.40	1.03-1.90	**0.03**	1.37	1.01-1.88	**0.05**	1.99	1.54-2.58	**<0.001**	1.82	1.40-2.38	**<0.001**	2.12	1.67-2.69	**<0.001**	2.07	1.63-2.63	**<0.001**
> 55	2.14	1.40-3.26	**<0.001**	2.17	1.41-3.35	**0.01**	3.13	2.17-4.54	**<0.001**	2.74	1.88-4.02	**<0.001**	3.17	2.28-4.41	**<0.001**	3.00	2.16-4.20	**<0.001**
**Time since smoking cessation***																		
1-10	0.99	0.61-1.60	0.96	1.25	0.77-2.06	0.36	0.82	0.55-1.23	0.345	1.11	0.73-1.68	0.61	0.91	0.62-1.34	0.63	1.17	0.79-1.74	0.43
11-20	0.64	0.42-0.98	**0.04**	0.77	0.50-1.19	0.24	0.67	0.47-0.95	**0.02**	0.78	0.54-1.12	0.18	0.73	0.53-1.03	0.07	0.89	0.64-1.26	0.51

21-30	0.56	0.36-0.86	**0.01**	0.63	0.40-0.99	**0.04**	0.79	0.56-1.12	0.19	0.82	0.58-1.18	0.28	0.80	0.58-1.10	0.16	0.91	0.66-1.25	0.55
> 30	Ref			Ref			Ref			Ref			Ref			Ref		
**Number of drinks (15.6ml) per day***																		
0-0.9 drinks	Ref			Ref			Ref			Ref			Ref			Ref		
1-2.9 drinks	1.44	1.06-1.96	**0.02**	1.53	1.12-2.09	**0.01**	1.66	1.30-2.13	**<0.001**	1.84	1.43-2.36	**<0.001**	1.15	0.91-1.44	0.23	1.21	0.96-1.52	0.11
3-4.9 drinks	2.13	1.49-3.04	**<0.001**	2.63	1.83-3.79	**<0.001**	2.15	1.59-2.88	**<0.001**	2.45	1.81-3.30	**<0.001**	1.63	1.26-2.11	**<0.001**	1.79	1.38-2.32	**<0.001**
≥ 5 drinks	3.22	2.35-4.44	**<0.001**	4.48	3.30-6.26	**<0.001**	3.66	2.80-4.81	**<0.001**	5.03	3.79-6.70	**<0.001**	2.72	2.15-3.44	**<0.001**	3.16	2.48-4.04	**<0.001**
**Age at drinking initiation**																		
Never drinker	Ref			Ref			Ref			Ref			Ref			Ref		
≤ 15	1.02	0.68-1.55	0.92	0.72	0.47-1.13	0.14	1.37	0.91-2.11	0.14	1.00	0.65-1.55	0.99	1.13	0.80-1.62	0.48	1.06	0.74-1.53	0.75
16-20	0.88	0.58-1.37	0.58	0.67	0.43.1.05	0.08	1.01	0.67-1.57	0.95	0.97	0.64-1.52	0.91	1.04	0.73-1.49	0.85	0.94	0.66-1.36	0.74
> 20	0.81	0.49-1.32	0.39	0.74	0.44-1.23	0.23	0.78	0.48-1.27	0.30	0.90	0.55-1.48	0.66	0.82	0.55-1.23	0.34	0.76	0.51-1.15	0.19

**Model adjusted for sex, age and ethnicity. ***Model with fixed effect of study center and adjusted for sex, age and ethnicity.

Hosmer-Lemeshow test (p-value): [1] = 0.818, [2] = 0.422, [3] = 0.502, [4] = 0.665, [5] = 0.206, [6] = 0.548

Supplementary Table 3. Analysis of 2,143 former smokers with HNSCC by subsite and geographic location with logistic regression – multiple models (INHANCE Consortium; 1984-2009).

		Oral cavity cancer			Oropharyngeal cancer			Laryngeal cancer		
		OR	IC 95%		OR	IC 95%		OR	IC 95%	
			Lower	Upper		Lower	Upper		Lower	Upper
Central Europe	**Tobacco (pack-years)**									
	0.1-10							1.00	1.00	1.00
	11-30							3.6	1.9	7.3
	31-50							6.7	3.4	14
	> 50							10	5.3	20.4
	**Age at smoking cessation (years)**									
	≤ 45							1.00	1.00	1.00
	46-55							2.6	1.7	4.1
	> 55							3.6	2.2	5.7
	**Time since smoking cessation (years)**							1.00	1.00	1.00
	1-10							2.6	1.7	4.1
	11-20							3.6	2.2	5.7
	21-30							1.4	0.8	2.5
	> 30							1.00	1.00	1.00
	**Number of drinks per day**									
	0 – 0.9 drinks							1.00	1.00	1.00
	1 – 2.9 drinks							0.8	0.5	1.3
	3 – 4.9 drinks							1.3	0.8	2.1
	≥ 5 drinks							1.7	1	2.9
Western/Southern Europe	**Tobacco (pack-years)**									
	0.1-10	1.00	1.00	1.00	1.00	1.00	1.00	1.00	1.00	1.00
	11-30	1.6	1.1	2.6	2.0	1.4	2.8	3.3	2.3	4.7
	31-50	4.7	3.3	8.6	4.8	3.4	7.0	6.7	4.7	9.8
	> 50	5.3	2.8	7.9	4.9	3.3	7.3	6.5	4.4	9.7

	**Age at smoking cessation (years)**									
	≤ 45	1.00	1.00	1.00	1.00	1.00	1.00	1.00	1.00	1.00
	46-55	3.7	2.5	5.5	4	3.1	5.3	3.9	3	5.1
	> 55	8.1	5.2	12.9	5.6	4	7.9	7.2	5.5	10
	**Time since smoking cessation (years)**									
	1-10	6.1	3.3	12.7	5.3	3.2	9.1	8.6	5.3	14.9
	11-20	1.7	0.8	3.6	2.5	1.5	4.4	3.8	2.3	6.6
	21-30	0.7	0.3	1.8	1.4	0.8	2.6	1.7	0.9	3.1
	> 30	1.00	1.00	1.00	1.00	1.00	1.00	1.00	1.00	1.00
	**Number of drinks per day**									
	0 – 0.9 drinks	1.00	1.00	1.00	1.00	1.00	1.00	1.00	1.00	1.00
	1 – 2.9 drinks	1.8	1.1	2.8	1.8	1.3	2.6	1.4	1	1.9
	3 – 4.9 drinks	2.5	1.5	4.2	2.6	1.8	3.8	2.3	1.7	3.3
	≥ 5 drinks	4.8	3.0	7.8	4.3	3.0	6.1	4.9	3.6	6.7
**North America**	**Tobacco (pack-years)**									
	0.1-10	1.00	1.00	1.00	1.00	1.00	1.00	1.00	1.00	1.00
	11-30	2.3	1.3	4.1	1.2	0.9	1.8	2.1	1.3	3.5
	31-50	2.9	1.6	5.4	2.1	1.4	3.1	3.6	2.1	6
	> 50	4.7	2.6	8.6	3.8	2.5	5.8	6.5	4	10.9
	**Age at smoking cessation (years)**									
	≤ 45	1.00	1.00	1.00	1.00	1.00	1.00	1.00	1.00	1.00
	46-55	1.9	1.2	3.1	1.6	1.1	2.2	2.1	1.4	3
	> 55	4.2	2.3	7.6	5.2	3.2	8.4	3.6	2.4	5.6
	**Time since smoking cessation (years)**									
	1-10	3.7	2.0	7.4	2.2	1.5	3.4	3.2	2.0	5.1
	11-20	2.1	1.1	4.4	0.8	0.5	1.4	1.4	0.8	2.2
	21-30	1.1	0.5	2.3	0.9	0.6	1.5	1.2	0.7	2.0
	> 30	1.00	1.00	1.00	1.00	1.00	1.00	1.00	1.00	1.00

	**Number of drinks per day**									
	0 – 0.9 drinks	1.00	1.00	1.00	1.00	1.00	1.00	1.00	1.00	1.00
	1 – 2.9 drinks	1.6	0.9	2.9	1.5	1.1	2.2	1.6	1	2.5
	3 – 4.9 drinks	4.4	2.4	8.2	2.5	1.5	4.0	2.4	1.4	4.1
	≥ 5 drinks	5.9	3.4	10.2	4.4	3.0	6.5	5.3	3.5	8.1
	**Tobacco (pack-years)**									
	0.1-10	1.00	1.00	1.00	1.00	1.00	1.00	1.00	1.00	1.00
	11-30	3.4	1.7	7.2	1.2	0.4	4.1	1.9	1	3.9
	31-50	5.1	2.5	11.1	3.5	1.2	11.6	3.6	1.8	7.3
	> 50	5.2	2.6	11.3	6.2	2.4	19.3	2.6	1	5.3
	**Age at smoking cessation (years)**									
	≤ 45	1.00	1.00	1.00	1.00	1.00	1.00	1.00	1.00	1.00
	46-55	2.3	1.4	3.8	4	1.9	8.3	2.9	1.7	4.8
	> 55	5.7	3.3	10.1	7.8	3.2	19.7	5.3	3	9.4
**South America**	**Time since smoking cessation (years)**									
	1-10	8.0	3.7	19.6	5.6	1.8	24.5	6.1	3.0	13.3
	11-20	4.0	1.7	10.0	2.3	0.6	10.9	3.2	1.5	7.2
	21-30	1.8	0.7	5.2	2.0	0.5	10.1	1.6	0.6	4.1
	> 30	1.00	1.00	1.00	1.00	1.00	1.00	1.00	1.00	1.00
	**Number of drinks per day**									
	0 – 0.9 drinks	1.00	1.00	1.00	1.00	1.00	1.00	1.00	1.00	1.00
	1 – 2.9 drinks	0.9	0.6	1.3	4.6	1.2	15.0	1.4	0.8	2.5
	3 – 4.9 drinks	3.4	1.6	7.3	6.3	1.4	20.0	2.1	0.9	4.7
	≥ 5 drinks	3.5	2.1	5.8	16.6	6.2	25.0	2.2	1.3	3.7

Central Europe corresponding Slovakia, Romania, Hungary, Poland, Germany and Russia. Western/Southern Europe corresponding Italy and France. All models were adjusted for sex and age.
